# Subjective Outcome Evaluation and Factors Related to Perceived Effectiveness of the Project P.A.T.H.S. in Hong Kong

**DOI:** 10.1100/2012/490290

**Published:** 2012-04-01

**Authors:** Daniel T. L. Shek, Lu Yu, Vicky Y. T. Ho

**Affiliations:** ^1^Department of Applied Social Sciences, The Hong Kong Polytechnic University, Hong Kong; ^2^Public Policy Research Institute, The Hong Kong Polytechnic University, Hong Kong; ^3^Kiang Wu Nursing College of Macau, Macau, China; ^4^Department of Sociology, East China Normal University, Shanghai 200241, China; ^5^Division of Adolescent Medicine, Department of Pediatrics, Kentucky Children's Hospital, University of Kentucky College of Medicine, Lexington, KY 40506-9983, USA

## Abstract

Based on a sample of 24,457 participated students, the present study investigated participants' subjective evaluation of the Tier 2 Program of the Project P.A.T.H.S. in the 2009/2010 academic year. Participants generally held positive views toward the Tier 2 Program and program instructor and perceived the program to be beneficial to their development. Programs involving adolescents alone were evaluated more positively than programs involving parents and/or teachers. Students' grade and program type did not show significant impact on participants' subjective evaluation of the project. Consistent with previous reports, perceived effectiveness of the program was significantly predicted by students' perceptions about the program and program instructor. These findings provide further support that the Tier 2 Program is effective in promoting positive development among adolescents with greater psychosocial needs.

## 1. Introduction

The Project P.A.T.H.S. is a large-scale positive youth development program designed for junior secondary school students in Hong Kong. The word “P.A.T.H.S.” denotes Positive Adolescent Training through Holistic Social Programmes. The aims of the project are to promote the holistic development of adolescents through nurturing their abilities, hence enhancing intrapersonal protective factors in them. There are two tiers of programs in the project. The Tier 1 Program adopts a universal prevention strategy targeting at all students joining the program regardless of their risk status. With the use of a structured curriculum-based approach, students learn competencies related to 15 positive youth development constructs including bonding, resilience, social competence, emotional competence, cognitive competence, behavioral competence, moral competence, self-determination, spirituality, self-efficacy, clear and positive identity, beliefs in the future, recognition for positive behavior, prosocial involvement, and prosocial norms [1]. 

In contrast to the Tier 1 Program, the Tier 2 Program adopts a selective prevention approach which is specifically designed for students with greater psychosocial needs in different psychosocial domains. Students in the Tier 1 Program who are identified by teachers or parents to have greater psychological needs are invited to participate in the Tier 2 Program. In view of the diverse needs of the students and to create more flexibility for the workers, the nongovernmental organizations (NGOs), who assist with the overall coordination and implementation of the project, would design appropriate programs that target the needs of the students based on the 15 positive youth development constructs, goals, and objectives proposed in this project. Several commonly used Tier 2 Program types include mentorship programs, mental health promotion programs, adventure-based counseling, parenting programs, service learning programs, and resilience enhancement programs [2]. Normally speaking, there are about one-fifth of the adolescents and/or their parents from the Tier 1 Program participating in the Tier 2 Program. While various evaluation findings have demonstrated that the Tier 1 Program has been successfully implemented, evidence that supports the effectiveness of the Tier 2 Program on adolescents with special psychosocial needs is relatively limited. 

As mentioned, school social work organizations have the flexibility to design the program according to different students' needs for the Tier 2 Program. As a result, different programs with varied content and goals were devised and implemented. This creates difficulty for conducting standardized objective evaluation of the Tier 2 Program. Against this background, a subjective outcome evaluation method was employed to examine the implementation and effectiveness of the Tier 2 Program. Subjective evaluation has been widely adopted in the education field, for example, using students' evaluation as a means to measure teaching effectiveness. Students' direct exposure to both the instructor and the instructing methods puts them in the best position to judge whether the instructional methods facilitate their learning [3]. According to Chen and Hoshower [4], students' subjective evaluation of teaching effectiveness can provide feedback on improving teaching, course content and structure, and examining issues such as development and validity of the evaluation instrument. Compared to objective evaluation methods, subjective evaluation gives a more detailed and comprehensive account of the subject being evaluated [5]. Concerning the possibility of the evaluator's bias in subjective evaluation, evidence shows support for both validity and reliability of students' subjective rating as a measure of teaching effectiveness [6, 7]. For the Project P.A.T.H.S., both subjective evaluation and objective evaluation have been used to understand the effectiveness of the program. The results obtained from the two evaluation approaches were convergent [8–11], which suggests that subjective evaluation is a feasible and reliable method in evaluating the project. 

There are three purposes of this study. The first purpose of the present study was to assess the effectiveness of the Tier 2 Program based on the subjective evaluation by a large sample of secondary school students in Hong Kong. The second purpose of this study was to identify factors that may affect participants' subjective evaluation of the Tier 2 Program. The first factor under study was program type. Based on previous classification of program content [12], four major program types were identified. These included (1) Type A—an approach that combines adventure-based counseling (ABC) and volunteer training and service (VTS), (2) Type B—adventure-based counseling (ABC) only, (3) Type C—volunteer training and service (VTS) only, and (4) Type D—other approaches without elements of ABC or VTS. While different types of program have different focuses, strengths, and weaknesses, it is possible that perceived program effectiveness by the participants varies across program types. To examine the relationship between program type and participants' subjective evaluation would help to identify the most desirable program by the participants and contribute to future youth program development. 

A second factor that might influence perceived effectiveness of the program was the type of participants. Due to various program designs, types of participant involved in different Tier 2 programs were not the same. There are programs designed exclusively for students, programs that involved both parents and students (such as family camps or parents talk), and programs requiring the participations of students, parents, and teachers. Literature has suggested a positive relationship between the involvement of significant people of adolescents in youth program and program effectiveness. For example, parental involvement has long been suggested as improving the outcomes of youth program [13]. Results of a recent alcohol prevention program showed that working through parents was an effective strategy to control youth drinking and delinquent behaviors [14]. It has also been suggested that the involvement of a mentor in youth development program is favorable. Through clear instructions and serving as role models and advocates, a mentor could enhance the social well-being and cognitive skills of the adolescents and promote their positive identity development [15]. Besides, teachers' support increased students' motivation and active engagement in prevention/intervention programs [16]. Therefore, it was expected that programs involving adolescents and parents as well as teachers would have better subjective outcomes than programs with only adolescents' participation. In this study, students' perceptions regarding programs with different participant types were compared.

Successful program implementation is also determined by the effectiveness of the instructors [17]. One study that evaluated a distant education program found that it was the instructor effectiveness (quality of instructor and instruction) instead of the computer technology that predicted students' perceived satisfaction of the online course [18]. While delivering program content to the participants, instructors act as the bridge between the program and its recipients. Researchers proposed that instructors who have high but attainable expectations and who provide encouraging and nonjudgmental feedbacks contribute to program participants' growth [19]. In evaluating the Tier 1 Program of the Project P.A.T.H.S., quite a few studies have shown that instructors' effective classroom management skills, familiarity with students, use of interactive delivery method, opportunity for reflection, good time management, and lesson preparation are crucial factors that influence program implementation quality [20–23]. It would be interesting to examine whether participants' evaluation of instructors in the Tier 2 Program would also predict the effectiveness of the program based on participants' subjective outcome evaluation. Besides, students' perceptions and interests about the program also contribute to effective program implementation [20]. Programs that were perceived positively by the participants often led to desirable short-term and long-term outcomes [2, 22, 24, 25]. While there were findings showing that participants' views towards the Tier 1 program significantly predicted program success, few studies have examined this relationship in the implementation of Tier 2 Program. As such, the third purpose of this study was to investigate how students' views on the Tier 2 Program and instructors may be related to their perceived effectiveness of the program.

To sum up, the present study attempted (1) to examine the profiles of subjective outcome evaluation findings of the Tier 2 Program conducted in 2009/10 school year based on a large sample of secondary school students, (2) to investigate the influence of participant type and program type on students' subjective evaluation of the program, and (3) to examine whether students' views on program and instructors predict their perceived effectiveness of the program.

## 2. Methods

### 2.1. Participants and Procedures

In 2009/2010 school year, a total of 42,771 participants from 231 schools joined the Tier 2 Program of the Project P.A.T.H.S., including 15,447 participants from Secondary 1, 13,569 from Secondary 2, and 13,755 from Secondary 3. Among these participants, 26,649 were core participants who were identified as having greater psychosocial needs by teachers, parents, and/or via self-administered questionnaires, and 3,378 were their parents and teachers. The remaining 12,744 participants were students who were not identified as the target participants of the Tier 2 program but also participated in the program, their parents, and teachers. 

The participating students were invited to respond to the Subjective Outcome Evaluation Form (Form C) immediately after completion of the Tier 2 Program. A total of 24,457 students (*M* = 42.39 students per school, ranging from 6 to 289) responded to Form C and the overall response rate was 91.78%. 

At the beginning of data collection, the purpose of the evaluation was explained, and the principle of confidentiality was repeatedly emphasized to the participants. The participants were asked to indicate their wish if they did not want to respond to the evaluation questionnaire (i.e., “passive” informed consent were obtained). Participants responded to all scales in the evaluation form in a self-administration format. Adequate time was provided for the participants to complete the questionnaire. To facilitate the program evaluation, the Research Team developed an evaluation manual with standardized instructions for collecting the subjective outcome evaluation data. In addition, during a 20-hour training workshop, adequate training was provided to the social workers who administered the survey on how to collect and analyze the data collected using the Form C.

### 2.2. Instruments

The Subjective Outcome Evaluation Form (Form C) was used to measure participants' perceptions of Tier 2 Program [26]. There are seven parts in this evaluation form.

- Participants' perceptions of the program, such as program design, quality of service, appropriateness of the program, and interaction among the participants (8 items).
- Participants' perceptions of the workers, such as the preparation of the workers, professional attitude and knowledge, and interaction with the participants (8 items).
- Participants' perception of the effectiveness of the program, such as promotion of different psychosocial competencies, resilience, and overall personal development (8 items).
- Things that the participants appreciated most (open-ended question).
- Opinion about the workers (open-ended question).
- Things that the participants learnt from the program (open-ended question).
- Areas that require improvement (open-ended question).

The present study focused on findings pertinent to the participants' views on the program, views on the program instructors, and perceived effectiveness of the program. After collecting the data, the social work service providers were requested to input the data in an EXCEL file developed by the Research Team which would automatically compute the frequencies and percentages associated with the different ratings for an item. When the providers submitted the reports, they were also requested to submit the softcopy of the consolidated data sheets. The data from all service providers were then aggregated to “reconstruct” the overall profile by the Research Team. Psychometric properties of the measures on the present sample can be seen in Table 1.

### 2.3. Data Analysis

First, descriptive statistics were employed to describe the profiles on the subjective evaluation of the program. Second, multivariate analysis of variance (MANOVA) was conducted to investigate whether the subjective evaluation of the program differed by program type and participant type. In the analyses, program type and participant type were independent variables with student grade included to control for its possible effect. Dependent variables were the three outcome evaluation measures, including participants' views about program, views about instructors, and perceived program effectiveness. Third, to examine how participants' views towards the program and the instructor may contribute to their subjective evaluation of program effectiveness, multiple regression analysis was performed, with views on program and instructor treated as the independent variables and perceived program effectiveness treated as the dependent variable.

## 3. Results

### 3.1. Descriptive Profile of the Perception of the Tier 2 Program

The descriptive profile for the Tier 2 Program implemented in 2009/2010 academic year is summarized in Table 2, which includes information about participant number, program attendance, and number of program aims and constructs as well as the mean overall effectiveness. As can be seen from Table 2, Type A program (ABC plus VTS) was the most widely employed approached, followed by Type B (ABC only), and then Type C (VTS only) and D (approaches other than ABC or VTS). Out of 575 programs, Type A was used in 240 programs, and Type B was adopted in 211 programs. A total of 210 programs involved only students, 41 involved both students and parents, 234 involved both students and teachers, and 89 involved students, parents, and teachers. The number of participants for each program ranged from 41.03 to 89.17, with an average program attendance rate ranged from 79.34% to 90.40%. The overall program effectiveness ranged from 4.15 to 4.76 on a six-point Likert scale on the positive side. 

The participants' views towards the program and numbers and percentages of participants who reported positive ratings (i.e., rating of 4 or above on a 6-point scale) on the three outcome evaluation measures are summarized in Table 3. As can be seen in the table, 99.7% of the respondents had positive views on the program. Specifically, 99.5% of the participants were satisfied with the service, and 99.7% of the participants would recommend others to participate in the program. Second, almost all participants had positive views towards the instructors. For example, 99.8% of the respondents felt that the workers understood the needs of the participants, and all respondents expressed satisfaction with the service. Third, 99.7% of the participants perceived the program as effective in different aspects. For example, 99.5% of the participants indicated that the program facilitated their growth and reported positive changes after joining the program. Besides, mean scores of the three scales for different types of programs by participants in different grades are presented in Table 4. All scores were above 4 on a six-point Likert scale towards the positive side, which further suggests that different types of program were generally perceived positively by students at different grades.

### 3.2. Effect of Participant Type, Program Type, and Student Grade on Participants' Perception of Tier 2 Program

The results of MANOVA showed significant main effect for participant type (Wilk's Λ = 0.96, *F*(9,1280) = 2.594, *P* < 0.01), indicating that type of participants involved in the program was significantly related to participants' ratings of the program. Post hoc analyses using Tukey's test were performed to further identify mean differences across groups. For participants' view on the program, programs involving students only were rated more favorably than programs that also involved parents and teachers (*P* < 0.05). For participants' view on program instructors, instructors were rated more favorably by participants from student-only programs than students from programs that also involved parents (*P* < 0.05) or both parents and teachers (*P* < 0.01). For perceived program effectiveness, participants from student-only programs rated the program as more effective than students from programs involving both students and parents (*P* < 0.05). The results of MANOVA showed that the effects of program type and grade of study were not significant (Wilk's Λ = 0.98, *F*(9,1280) = 1.30, *P* = 0.23). This suggests that participants' subjective evaluation of the program did not vary across program types and participant grades. 

### 3.3. Predictors of Perceived Program Effectiveness

Results of regression analyses showed that participants' view on program significantly predicted program effectiveness (*β* = 0.67, *P* = 0.00). In addition, participants' view on program instructor was also a significant predictor of their perceived effectiveness of the program (*β* = 0.26, *P* = 0.00). Together, approximately 83% in variability of participants' perceived effectiveness of the program was associated with their views on the program and program instructor (Table 5).

## 4. Discussion

The current study examined the Tier 2 Program of the Project P.A.T.H.S. in terms of participants' subjective evaluation of three domain measures, including views about the program, views about the instructors, and perceived effectiveness of the program. Results showed that students generally held positive views towards the program. Over 98% of the students viewed the program and instructors in favorable light. They regarded the program as of good quality and satisfying and perceived the program instructors as professional and caring. The program was considered as beneficial to their development. They agreed that the program promoted their growth and created positive changes in them. In line with previous evaluation findings of the project [2, 19, 20], the present results provide further support that the Tier 2 Program is effective in promoting positive development among adolescents with special psychosocial needs. 

Participants' subjective evaluation was compared across programs with different types of participants to examine whether the involvement of parents and/or teachers would enhance positive outcomes of the program. Inconsistent with previous findings [13, 15, 16], results of the present study showed that adolescents evaluated student-only programs more favorably than programs involving parents and/or teachers, in terms of views about the program, instructors, and perceived effectiveness of the program. There are several possible explanations for this interesting finding. First, there were different definitions of parental involvement used in studies in the literature. In the Project P.A.T.H.S., a program is classified as involving parents as long as it includes parent participants, while other studies considered parental involvement as exhibiting “good parenting at home” or “thorough going participation in school functions and school governance” [27]. Furthermore, as suggested by Desforgfes, there were “different measures of parental involvement even for a given definition” [27, page 14]. Thus, involvement of parents may not give a precise image about the degree of parental involvement. Second, the outcome measures used to evaluate program effectiveness varied across studies. In many program evaluation studies, adolescents' academic achievement and school behaviors were commonly used as outcome measures [28–32], with the subjective feelings of the participants being largely ignored. In contrast, the present study focused on participants' own preference and perceived effectiveness of the program. It is possible that a program exclusively focusing on students' academic achievement while overlooking students' psychological health may not be evaluated favorably by the participants. This may also contribute to the discrepancy between the present finding and the previous ones. Thirdly, student-only programs may be more interesting to adolescents than parent/teacher involved programs. In the Tier 2 Program, common parent/teacher-involved programs include parent talks and mentorship programs which are obviously less attractive to students than student-only programs like adventure camps, multiple intelligence training, city hunt and war games. Fourth, the current finding may reflect the fact that adolescents like to spend leisure time with peers rather than their parents or teachers. As suggested by Shek and Ma [33], adolescents' relationship with parents become tense as they seek more independence and when peer influence becomes increasingly prominent. Adolescents' desire to associate with peers leads them to spend more time with peers than with parents in leisure activities [34]. Despite differences in the ratings, it should be noted that adolescents generally viewed the programs and instructors positively and perceived the programs as beneficial to their development, regardless of the type of participants involved in the programs.

The present study also compared students' subjective evaluation based on program types. The nonsignificant effect suggests that different types of programs were perceived equally effective by the students, and that program type was not a key factor determining the effectiveness of the project. Adventure-based counseling (ABC) and volunteer training and service (VTS) were the two most popular programs adopted by program implementers. In the ABC program, participants were placed in a situation of unfamiliarity and were challenged to engage in activities that would lead to successful outcomes [9]. In this way, participants were able to experience positive behavioral, cognitive, and affective change [35–37]. The VTS program focused on helping adolescents to acquire a sense of responsibility and re-conceptualize the way in which they view the world through participating in volunteer training and services [38]. Both approaches are relatively mature technique in youth intervention programs and have shown favorable effects for at-risk adolescents [8, 9, 35–37]. The positive results obtained in the present study provide further support for the effectiveness of the two approaches. In addition, Type D program (programs involving components other than ABC and VTS) also received high ratings from the students. Future studies should further identify what other methods were used in Type D program and the key determinants of program success other than program types. 

Finally, the present study examined how participants' views on program and instructors were related to perceived effectiveness of the program. Results showed that both students' views on program and on instructors significantly predicted their subjective evaluation of the program effectiveness. Previous theories and reports have consistently shown that quality of program and program implementers are two determinants for program success [39, 40]. In a thorough review of literature across different areas of youth risk behaviors, Nation et al. [41] summarized nine characteristics of effective prevention program, most of which are directly related to high program quality or good program workers. For example, comprehensiveness of a program, theory-driven design, and social-cultural relevance are indicators of program quality, while appropriately timing, use of multiple teaching methods, and well-trained staff are all associated with competent program implementers. Echoing prior results, the present findings further highlight the importance of the two factors in effective youth program implementation. 

It was also found that students' perceptions about the program appeared to be a stronger predictor of program effectiveness as compared to views towards instructors. Similarly, while perceived usefulness of program was found to be significantly predicted by students' views towards the program, nonsignificant relationship between views on instructors and program success was reported in Shek and Ma's study [39]. They proposed that the ceiling effect due to the high score of perceived quality of instructors as compared to perceived program content and effectiveness may be a plausible source for the nonsignificant finding. This explanation may also apply to the relatively low correlation between ratings on instructors and program effectiveness in the present study as perceived quality of instructors did receive the highest score from the participants among the three subjective evaluation indictors. Another possibility may be that there are other variables moderating the effects of students' views towards instructors on their perceived effectiveness, for example, students' familiarity with the instructors. Further research is needed to examine this possible explanation. 

Despite the positive findings, three limitations of the present study should be noted. First, as the reports were “reconstructed” from the evaluation reports submitted by participating agencies using school as a unit, individual variations were lost in the process. Other evaluations of the Tier 2 Program using individual students as a unit should be carried out so as to understand individual participants' comments and perception of the program. However, previous findings showed that the pictures derived from these two strategies were more or less the same [42, 43]. Second, the utilization of subjective evaluation has its own limitation. Participants' evaluation of the program may be affected by their personal experience and may not entirely reflect the true outcome of the program. For example, a student may rate the program effectiveness based on positive experience during the program, such as having made new friends or developed positive relationship with instructors, rather than true program quality. As such, objective outcome evaluation of the Tier 2 Program should be carried out to address such limitation. Third, besides subjective evaluation findings, other evaluation data utilizing qualitative methods should be used. When this strategy is used, the subjective experiences of the program participants could be better understood.

In conclusion, the present study provided important information on the implementation of the Tier 2 Program of the Project P.A.T.H.S. during the 2009/2010 academic year. Aligned with previous evaluation findings, results of this study suggest that the Tier 2 Program is beneficial to the development of youth with higher psychosocial needs. Taken as a whole, the available findings suggest that the Project P.A.T.H.S. is an effective positive youth development program to promote holistic development of Chinese adolescents in Hong Kong [44–46].

## Figures and Tables

**Table 1:** Psychometric properties of the subjective outcome evaluation measures.

	Cronbach's **α**	Mean interitem correlation	Interscale correlation	
			Views about the instructor	Perceived program effectiveness
Views about the program	0.98	0.88	0.93	0.91
Views about the instructor	0.99	0.91	—	0.88
Perceived program effectiveness	0.99	0.90	—	—

**Table 2:** Summary of program characteristics and effectiveness.

Main program approach	Clientele	Average no. of participants	Average program attendance (%)	Average no. of program aims indicated in the reports	Average no. of constructs indicated in the reports	Mean of overall effectiveness
Adventure-based counseling approach and volunteer training and services (Type A) (*N* = 240)	a (*N *= 100)	43.58	80.95	2.70	5.89	4.75
	b (*N* = 17)	63.59	79.34	2.47	6.41	4.38
	c (*N* = 74)	41.03	86.51	2.51	7.19	4.72
	d (*N* = 49)	77.47	81.50	2.39	7.61	4.69

Adventure-based counseling approach only (Type B) (*N* = 211)	a (*N* = 57)	42.51	84.85	2.81	5.67	4.69
	b (*N* = 17)	54.71	81.01	2.59	7.18	4.76
	c (*N* = 111)	53.79	87.29	2.32	7.81	4.69
	d (*N* = 26)	86.62	81.83	2.92	7.73	4.51

Volunteer training and services only (Type C) (*N* = 57)	a (*N* = 29)	41.24	84.05	2.86	6.28	4.66
	b (*N* = 3)	72.00	84.63	3.00	7.67	4.66
	c (*N* = 17)	52.35	86.42	2.71	9.53	4.58
	d (*N* = 8)	60.87	81.07	2.75	7.00	4.57

Other approaches (Type D) (*N* = 66)	a (*N* = 24)	36.13	88.73	2.33	4.33	4.74
	b (*N* = 4)	60.00	84.73	2.75	5.00	4.15
	c (*N* = 32)	54.66	84.36	2.66	7.19	4.75
	d (*N* = 6)	89.17	90.40	1.67	5.17	4.26

Note: a = only students involved, b = students and parents involved, c = students and teachers involved, d = students, parents and teachers involved.

**Table 3:** Participants' positive ratings on the three outcome evaluation measures.

	*n * (positive view)*	%	*n * (negative view)*	%	*n * (total responses)
*Participants' view on the program*					
(1) The activities were carefully planned.	29840	99.4	195	0.6	30035
(2) The quality of the service was high.	29425	98.8	367	1.2	29792
(3) The service provided could meet the participants' needs.	29500	99.3	196	0.7	29696
(4) The service delivered could achieve the planned objectives.	29930	99.5	142	0.5	30072
(5) Participants could get the service they wanted.	29493	99.7	91	0.3	29584
(6) Participants had much interaction with other participants.	29988	99.7	84	0.3	30072
(7) Participants would recommend others who have similar needs to participate in the program.	29913	99.6	110	0.4	30023
(8) On the whole, participants were satisfied with the service.	29872	99.7	97	0.3	29969
Total mean score	29930	99.5	142	0.5	30072

*Participants' view on the program instructor*					
(1) The worker(s) had professional knowledge.	29954	99.6	118	0.4	30072
(2) The worker(s) demonstrated good working skills.	30111	100	0	0	30111
(3) The worker(s) were well prepared for the program.	30003	99.8	69	0.2	30072
(4) The worker(s) understood the needs of the participants.	30003	99.8	69	0.2	30072
(5) The worker(s) cared about the participants.	30111	100	0	0	30111
(6) The worker(s)' attitudes were very good.	29588	99.8	69	0.2	29657
(7) The worker(s) had much interaction with participants.	29758	100.0	0	0	29758
(8) On the whole, participants were satisfied with the worker(s).	30111	100.0	0	0	30111
Total mean score	30003	100.0	0	0	30003

*Participants' perceived effectiveness of the program *
(1) The service has helped participants a lot.	29904	99.6	123	0.4	30027
(2) The service has enhanced participants' growth.	29988	99.6	123	0.4	30111
(3) In the future, participants would receive similar service(s) if needed.	29398	98.6	429	1.4	29827
(4) Participants have learnt how to help themselves through participating in the program.	30003	99.6	108	0.4	30111
(5) Participants have had positive change(s) after joining the program.	29954	99.5	157	0.5	30111
(6) Participants have learnt how to solve their problems through participating in the program.	29954	99.7	88	0.3	30111
(7) Participants' behavior has become better after joining this program.	29923	99.5	145	0.5	30068
(8) Those who knew the participants agree that this program has induced positive changes in them.	29847	99.1	264	0.9	30111
Total mean score	29960	99.6	108	0.4	30068

Note: *positive view = rating of 4 or above on a 6-point scale; negative view = rating below 4 on a 6-point scale.

**Table 4:** Mean score of views on program, views on instructor, and perceived program effectiveness for participants at different grades and in schools adopting different program approaches.

		View on program	View on instructor	Program effectiveness
Grade	1	4.70	4.86	4.67
	2	4.71	4.89	4.67
	3	4.75	4.93	4.69
Program type	A	4.75	4.92	4.70
	B	4.69	4.87	4.67
	C	4.68	4.84	4.62
	D	4.71	4.89	4.68

Each item was rated on a 6-point Likert scale, with scores of 4 and above meaning positive views.

**Table 5:** Linear regression analyses predicting positive views towards the Tier 2 program effectiveness.

	Program effectiveness	
	*β* ^a^	*R* ^2^
View on program	0.668	.836
View on instructor	0.259	

^a^Standardized coefficients, **P = 0.00.
